# Violence against children during the COVID-19 pandemic

**DOI:** 10.2471/BLT.20.283051

**Published:** 2021-08-13

**Authors:** Amiya Bhatia, Camilla Fabbri, Ilan Cerna-Turoff, Ellen Turner, Michelle Lokot, Ajwang Warria, Sumnima Tuladhar, Clare Tanton, Louise Knight, Shelley Lees, Beniamino Cislaghi, Jaqueline Bhabha, Amber Peterman, Alessandra Guedes, Karen Devries

**Affiliations:** a Department of Global Health and Development, London School of Hygiene and Tropical Medicine, 15-17 Tavistock Pl, London WC1H 9SH, England.; b Department of Social Work, University of the Witwatersrand, Johannesburg, South Africa.; c Child Workers in Nepal (CWIN), Kathmandu, Nepal.; d Harvard T.H. Chan School of Public Health, FXB Center for Health and Human Rights, Cambridge, United States of America (USA).; e Department of Public Policy, University of North Carolina, Chapel Hill, USA.; f UNICEF Office of Research Innocenti, Florence, Italy.

## Abstract

The coronavirus disease 2019 (COVID-19) pandemic has affected children’s risk of violence in their homes, communities and online, and has compromised the ability of child protection systems to promptly detect and respond to cases of violence. However, the need to strengthen violence prevention and response services has received insufficient attention in national and global pandemic response and mitigation strategies. In this paper, we summarize the growing body of evidence on the links between the pandemic and violence against children. Drawing on the World Health Organization’s INSPIRE framework to end violence against children, we illustrate how the pandemic is affecting prevention and response efforts. For each of the seven INSPIRE strategies we identify how responses to the pandemic have changed children’s risk of violence. We offer ideas for how governments, policy-makers, and international and civil society organizations can address violence in the context of a protracted COVID-19 crisis. We conclude by highlighting how the current pandemic offers opportunities to improve existing child protection systems to address violence against children. We suggest enhanced multisectoral coordination across the health, education, law enforcement, housing, child and social protection sectors. Actions need to prioritize the primary prevention of violence and promote the central role of children and adolescents in decision-making and programme design processes. Finally, we stress the continued need for better data and evidence to inform violence prevention and response strategies that can be effective during and beyond the COVID-19 pandemic.

## Résumé

La pandémie de maladie à coronavirus 2019 (COVID-19) a eu un impact sur le risque de violence à l'égard des enfants à domicile, au sein de leur communauté et en ligne. Elle a également empêché les systèmes de protection de l'enfance d'identifier rapidement les situations de ce type et d'y réagir dès que possible. Pourtant, la nécessité de renforcer les services de prévention et d'action en la matière n'a pas été suffisamment prise en compte dans les stratégies nationales et internationales d'intervention et d'atténuation des effets de la pandémie. Le présent document reprend l'accumulation de preuves confirmant les liens entre pandémie et violence à l'égard des enfants. En nous inspirant du cadre INSPIRE de l'Organisation mondiale de la Santé visant à mettre fin à la violence à l'encontre des enfants, nous illustrons la façon dont la pandémie affecte les efforts de prévention et d'action. Pour chacune des sept stratégies INSPIRE, nous déterminons comment les mesures de lutte contre la pandémie ont influencé le risque de violence envers les enfants. Nous formulons des pistes pour que les gouvernements, les législateurs, les institutions internationales et les organisations de la société civile puissent remédier à cette violence dans un contexte de crise prolongée due à la COVID-19. En guise de conclusion, nous mettons en lumière les opportunités qu'offre la pandémie actuelle d'améliorer les systèmes existants de protection de l'enfance pour mieux combattre la violence envers les enfants. Nous suggérons d'accroître la collaboration entre les secteurs de la santé, de l'éducation, du maintien de l'ordre, du logement, des droits de l'enfant et de la protection sociale. Les actions entreprises doivent se focaliser sur la prévention primaire de la violence et promouvoir le rôle central des enfants et adolescents dans les processus de conception de programmes et de prise de décisions. Enfin, nous soulignons le besoin permanent de données et de preuves fiables pour orienter les stratégies de prévention et d'intervention face à la violence, afin de garantir leur efficacité pendant et après la pandémie de COVID-19.

## Resumen

La pandemia de la enfermedad por coronavirus (COVID-19) ha afectado al riesgo de violencia infantil que sufren los niños en sus hogares, comunidades y en línea, y ha puesto en peligro la capacidad de los sistemas de protección infantil para detectar y responder rápidamente a los casos de violencia. Sin embargo, la necesidad de reforzar los servicios de prevención y respuesta a la violencia no ha recibido suficiente atención en las estrategias nacionales y mundiales de respuesta y mitigación de la pandemia. En este documento, resumimos el creciente conjunto de pruebas sobre los vínculos entre la pandemia y la violencia infantil. Basándonos en el marco INSPIRE de la Organización Mundial de la Salud para poner fin a la violencia infantil, ilustramos cómo la pandemia está afectando a los esfuerzos de prevención y respuesta. Para cada una de las siete estrategias de INSPIRE, identificamos cómo las respuestas a la pandemia han cambiado el riesgo de violencia infantil. Ofrecemos ideas sobre cómo los gobiernos, los responsables políticos y las organizaciones internacionales y de la sociedad civil pueden abordar la violencia en el contexto de una crisis prolongada de COVID-19. Concluimos destacando cómo la pandemia actual ofrece oportunidades para mejorar los sistemas de protección infantil existentes para abordar este tipo de violencia. Sugerimos una mayor coordinación multisectorial en los sectores de la salud, la educación, la aplicación de la ley, la vivienda y la protección social infantil. Las acciones deben priorizar la prevención primaria de la violencia y promover el papel central de los niños y adolescentes en los procesos de toma de decisiones y en el diseño de programas. Por último, subrayamos la necesidad permanente de contar con mejores datos y pruebas para fundamentar las estrategias de prevención y respuesta a la violencia que puedan ser eficaces durante la pandemia de COVID-19 y seguir vigentes cuando ésta pase.

## ملخص

لقد أثرت جائحة مرض فيروس كورونا 2019 (كوفيد 19) على مخاطر تعرض الأطفال للعنف في منازلهم ومجتمعاتهم وعبر الإنترنت، كما أخلت بقدرة أنظمة حماية الطفل على اكتشاف حالات العنف والاستجابة لها بسرعة. ومع ذلك، فإن الحاجة إلى دعم خدمات الوقاية من العنف والاستجابة له، لم تحظ باهتمام كاف في الاستراتيجيات الوطنية والعالمية للاستجابة للجائحة والتخفيف من حدتها. نقوم في هذه الورقة بتلخيص نلخص الحجم المتزايد من الأدلة على الروابط بين الوباء والعنف ضد الأطفال. وبالاعتماد على إطار عمل INSPIRE التابع لمنظمة الصحة العالمية لإنهاء العنف ضد الأطفال، نوضح كيف يؤثر الوباء على جهود الوقاية والاستجابة. بالنسبة لكل من استراتيجيات INSPIRE السبع، نحدد كيف أدت الاستجابات للوباء إلى تغيير خطر تعرض الأطفال للعنف. نحن نقدم أفكارًا حول كيفية قيام الحكومات، وواضعي السياسات، والمنظمات الدولية، ومنظمات المجتمع المدني، بالتعامل مع العنف في ظل أوضاع أزمة كوفيد 19 الممتدة. ونختتم الورقة بتسليط الضوء على كيفية توفير الوباء الحالي الفرص لتحسين أنظمة حماية الطفل الحالية للتعامل مع العنف ضد الأطفال. ونحن نقترح تعزيز التنسيق متعدد القطاعات عبر قطاعات الصحة والتعليم وإنفاذ القانون والإسكان والأطفال والحماية الاجتماعية. وتحتاج الإجراءات إلى إعطاء الأولوية للوقاية الأولية من العنف وتعزيز الدور المركزي للأطفال والمراهقين في عمليات صنع القرار وتصميم البرامج. وأخيرًا، نركز على الحاجة المتواصلة إلى بيانات وأدلة أفضل لتحسين استراتيجيات الوقاية من العنف والاستجابة له، والتي يمكن أن تكون فعالة أثناء جائحة كوفيد 19، وبعدها.

## 摘要

新型冠状病毒肺炎 (COVID-19) 大流行影响了儿童在家庭、社区和网络上遭受暴力的风险，削弱了儿童保护体系迅速发现暴力案件并做出应对的能力。不过，国家和全球流行病应对和缓解策略并没有对加强暴力预防和应对服务给予充分的重视。我们在本文中总结发现，越来越多的证据表明，这场大流行和针对儿童的暴力行为之间存在着联系。我们借鉴了世界卫生组织为消除针对儿童的暴力行为而提出的"INSPIRE" 框架，阐述了这场大流行如何影响预防和应对工作。对于 INSPIRE 七项策略中的每一项，我们都明确阐述了大流行应对工作是如何改变儿童遭受暴力行为风险的。我们为各国政府、政策制定者以及国际和民间社会组织如何在旷日持久的新型冠状病毒肺炎危机背景下解决暴力风险，提供了指导意见。我们强调了当前的大流行如何提供机会以完善现有的儿童保护体系，从而解决针对儿童的暴力问题。我们建议加强卫生、教育、执法、住房、儿童和社会保护部门之间的多部门协调工作。采取行动时应优先考虑暴力的初级预防，并且在制定决策和设计方案过程中以儿童和青少年权益为中心。最后，我们强调，需要持续收集更优质的数据和证据，为暴力预防和应对策略提供依据，从而令这些策略可以在新型冠状病毒肺炎大流行期间和之后持续保持有效。

## Резюме

Пандемия заболевания, вызванного коронавирусом в 2019 г. (COVID-19), повлияла на риск столкновения детей с насилием дома, в общественных местах и в Инернете и поставила под сомнение способность систем защиты детей своевременного выявления случаев насилия и реагирования на них. Однако в рамках национальной и международной реакции на пандемию и в соответствующих стратегиях по смягчению ее последствий уделяется недостаточно внимания необходимости укрепить службы, направленные на профилактику насилия и соответствующее реагирование. В данной статье авторы постарались подытожить растущий объем свидетельств связи между пандемией и насилием в отношении детей. Опираясь на рамочную стратегию ВОЗ по прекращению насилия над детьми (INSPIRE), они показывают, как повлияла пандемия на усилия по профилактике и реагированию. Для каждой из семи стратегий INSPIRE было определено, как реакция на пандемию изменила риски, связанные с насилием над детьми. Авторы выдвигают разного рода идеи относительно того, как правительства, лица, принимающие решения, а также международные и национальные гражданские организации могут реагировать на проблему насилия в свете продолжающегося кризиса, вызванного COVID-19. В заключение авторы подчеркивают те возможности, которые возникли в связи с пандемией в части улучшения существующих систем защиты детей и их реакции на проявления насилия в отношении детей. Авторы предлагают усилить взаимную координацию между секторами здравоохранения, образования, правоохранительными органами и органами, занимающимися вопросами жилищно-бытовых условий, защитой детей и социальной защитой. Среди действий на первое место следует поставить первичное предотвращение насилия и поощрение центральной роли детей и подростков в процессах принятия решений и разработки характера таких программ. Наконец, авторы подчеркивают, что по-прежнему существует необходимость в получении более качественных данных и доказательств, которые позволяли бы разрабатывать стратегии профилактики и реагирования на насилие, способные эффективно работать в условиях пандемии COVID-19 и не только.

## Introduction

Throughout the coronavirus disease 2019 (COVID-19) pandemic, children have often been referred to as silent spreaders, low-risk or invisible carriers of the disease. These descriptions negate the well-documented adverse effects of the COVID-19 pandemic on child health and well-being, including the increased risk of experiencing violence.^1^^,^^2^ Violence against children includes physical, sexual and emotional abuse, neglect, bullying, assault, homicide and sexual exploitation. Caregivers, adults, peers, teachers, law enforcers or strangers can perpetrate violence against children in public, private and institutional spaces.^3^^,^^4^ Ongoing responses to the pandemic have included restrictions on people’s movements and the closure of schools, services and businesses. Increases in violence against children and women can be linked to the COVID-19 pandemic and the associated response measures which have limited people’s access to health services and exacerbated economic insecurity.^5^^,^^6^ The pandemic has exposed and entrenched pre-existing social inequities in the prevalence of violence against children,^7^ and has highlighted important shortcomings in global and national violence prevention and response efforts. Evidence from past epidemics also indicates an increased risk of violence against children, affirming the important role that child safeguarding should play during, and beyond, protracted crises.^7^ Although the pandemic has drawn global attention to violence against women and children, prevention and response efforts continue to be underfunded and have received insufficient attention in COVID-19 strategies developed by governments and global organizations.

In this paper, we examine how the repercussions of the COVID-19 pandemic can increase the risk of violence against children and discuss how the pandemic offers lessons to improve violence prevention and response. We draw on the framework of *INSPIRE: seven strategies for ending violence against children* developed by the World Health Organization and partners.^4^ INSPIRE highlights how violence against children can occur across a wide range of settings and contexts. The framework includes evidence-based strategies to support countries to address violence against children and achieve the related sustainable development goals. The seven strategies include improving laws, social norms, children’s environments, caregiver support, social protection and safety nets, response services and education.^4^^,^^8^ We conclude with lessons for policy and practice to prevent and respond to the adverse effects of the COVID-19 pandemic on violence against children. Drawing on existing initiatives and recent innovations, we centre our recommendations in the principles of child rights and health equity, and on the importance of collaboration among organizations from different sectors.

## Current evidence

There is growing evidence of the consequences of the pandemic on violence against children.^5^^,^^6^^,^^9^ Despite increasing numbers of studies exploring risk factors and recent trends in violence against children, little attention has been given to violence prevention programming and policy or to mitigation and recovery strategies.^6^ A review in 2021 identified 48 studies published between March and December 2020 documenting the impact of the pandemic on violence against children.^5^ These studies primarily focused on children’s experiences of physical and psychological violence at home, with few studies examining sexual violence, adolescent intimate partner violence, bullying, and youth and community violence. With few exceptions, the evidence is from high-income countries. We highlight four findings from that review.^5^ First, the researchers found 15 studies analysing data from surveys conducted among parents and caregivers and four studies among service providers that consistently reported increases in family violence. Second, three studies that relied on hospital data found increases in violence-related injuries and evidence of child abuse. Third, 10 studies found calls to police or helplines increased in some countries and decreased in others. Finally, 10 studies analysing referrals to child protection services or to the police primarily found decreases in reported cases of violence against children. The authors hypothesized the decreases were linked to stay-at-home orders and the closure of schools and services, making it challenging for teachers, medical practitioners and social workers to identify cases of violence.^10^ Taken together, the evidence suggests that violence has both increased and become less visible.^9^ These findings are broadly consistent with the literature on the impact of the pandemic on violence against women, highlighting the connections between these forms of violence.^6^

## Addressing violence

Drawing on the seven strategies of the INSPIRE framework, we illustrate how the COVID-19 pandemic affected violence against children and provide examples of possible response strategies based on experiences from different countries and organizations (Table 1). The strategies to address violence against children vary by context and should be understood through an intersectional lens. Such an approach examines how children’s age, sex, gender identity, sexual orientation, race, ethnicity, socioeconomic status, migrant status and disability status affect their experiences and reporting of violence.^9^^,^^11^

**Table 1:** Policies and practices to address adverse effects of the COVID-19 pandemic on violence against children

INSPIRE strategy^a^	Adverse effects of the pandemic	Addressing violence against children in the context of the pandemic
Implementation and enforcement of laws	• Closure of courts and limited access to justice for children, including ongoing legal cases, restraining orders, custody hearings and juvenile justice • Separation of children from caregivers and family, with delays to family reunification • Limited monitoring of residential care institutions and detention centres	*Aim: Enhance access to justice for children*
		• Designate violence services as essential • Prioritize cases of violence that involve children • Conduct video and phone-based court hearings and child testimonies • Prioritize family reunification and efforts to ensure children separated from their caregivers have regular opportunities to communicate with them and receive care and support during the separation period • Prevent the detention of children • Protect the rights of children in detention and prioritize releasing children and women with children from custody
Norms and values	• Disruption of informal networks of support from extended family members, friends and neighbours • Increase in the influence of social norms that condone violence • Increased acceptability or justification for violence against children	*Aim: Change social norms that condone violence against children and sanction help-seeking*
		• Run public campaigns on help-seeking and prevention of violence against children • Increase the visibility of positive practices and attitudes to prevention and response to violence against children • Advocate with decision-makers, governments and agencies to prioritize continued funding of programmes addressing violence against children
Safe environments	• Loss of community-level or public safety nets and services (such as community centres, safe spaces and child-friendly spaces), particularly for children who were experiencing violence at home • Increase in alcohol and substance use in households • Use of police to enforce restrictions on movements • Increase in online bullying and exploitation	*Aim: Prevent police and gun violence and develop child-friendly cities*
		• Limit consumption of alcohol inside the household, including integration of substance abuse prevention with phone and online mental health services • Leverage existing after-school programmes and community-based activities to include most vulnerable groups of children (for example, girls’ clubs) • Limit access to firearms and other weapons • Prevent violent contact between young people and the police • Reduce militarization of law enforcement • Improve accountability of cases of violence against children perpetrated by law enforcement • Enhance safeguards and safety measures for online platforms • Develop child-friendly cities and public spaces in consultation with young people
Parent and caregiver support	• Increased stress and anxiety of caregivers • Changes in caring responsibilities due to economic changes and shifts in power dynamics within households	*Aim: Provide access to mental health and parenting services*
		• Ensure sick leave, parental leave and access to mental health services for caregivers • Ensure remote, culturally sensitive and gender responsive mental health provision and support to families coping with shifts in economic roles and financial strain • Provide resources and support for caregivers on parenting, approaches to nonviolent discipline (such as online dialogues, teacher–parent groups, community meetings) • Provide online and mobile-based parenting advice and counselling • Consider radio and television broadcasts to reach more remote areas
Income and economic strengthening	• Greater economic hardship, food insecurity, financial constraints and associated economic stressors, due to job loss, reduced household income and caregivers’ illness or death • Increased risk of child labour and sexual exploitation being used to supplement household income	*Aim: Provide child-sensitive social protection*
		• Create social protection systems and programmes to alleviate adverse economic impact on households and children (for example: child vouchers and child benefits; school meals for children; paid sick leave and parental leave; cash transfers prioritizing households with children; and universal basic income) • Expand and strengthen informal and community safety nets • Make emergency economic transfers and subsidies for households and employees adversely affected by COVID-19, with a specific focus on prioritizing households with children • Freeze eviction of families from their homes to prevent housing insecurity for households with children • Provide access to credit at low repayment rates, with delayed loan repayments by private and public institutions
Response and support services	• Disruptions in access to child protection services • Decreased reporting of violence due to school closures and reduced access to violence prevention and health services • Limited awareness among survivors of violence about in-person services and where and how to seek online or remote services • Closure or changes to residential care facilities for children	*Aim: Provide inclusive violence response services*
		• Designate child protection and violence response services as essential • Provide access to personal protective equipment for frontline staff and training to staff across different sectors on the increased risk of violence against children • Activate and strengthen child helpline services by leveraging mobile phone-based platforms • Integrate child-dedicated referral pathways within existing helpline services • Expand access to services within domestic violence shelters to include children • Include screening systems for child protection concerns within existing COVID-19 health services • Fund and support community-based organizations for violence prevention • Create non-traditional focal points for reporting violence (such as food distribution, pharmacies, community workers) • Mobilize youth networks and schools to share information about services • Train staff across different sectors to improve identification and referral for cases of violence against children • Use remote social work and case management services, including development of online platforms for free child protection services and psychological support • Establish family strengthening programmes and safe, family-based alternative care arrangements for children in residential care
Education and life skills	• Reduced identification of cases of child abuse and maltreatment via the education system • Increased bullying in online platforms • Lack of nutritional meals provided at school, especially for children who were beneficiaries of school meal programmes • Long-term challenges with access to school environments for children unable to access remote learning • Possible increase in school dropout	*Aim: Provide access to safe school environments*
		• Provide training and support for school staff to identify and refer children affected by violence remotely • Provide psychosocial support to children and families • Create and adapt safeguarding policies and staff training and induction in schools • Run back-to-school campaigns targeted at the most vulnerable groups of children • Provide relief from school fees for parents • Establish community food distribution systems • Adapt and implement remote and in-person school-based violence prevention programmes

COVID-19: coronavirus disease 2019.

^a^ Based on the INSPIRE strategy of the World Health Organization, 2018.^4^

### Laws

The COVID-19 response has affected access to justice for children who may have experienced violence. Many courts have offered limited services, which may have delayed children’s cases. Children waiting for custody, asylum decisions or family reunification may be separated from caregivers and families for extended periods or are obliged to stay in the same community with the perpetrators of violence while awaiting justice.^12^ To address the risks for children in contact with the law, courts have started to hold remote and online sessions to reinstate children’s access to the legal system. Courts should seek to prioritize family law cases; ensure emergency measures do not unlawfully restrict or suppress children’s rights; and allow exemptions from movement restrictions for children fleeing violence.^13^ COVID-19 containment measures have also compromised legal and advocacy efforts to ensure children are not in detention and to guarantee children in the juvenile justice system have timely access to lawyers.^14^ For children in conflict with the law, improvements should be made to the safety of detention facilities so child detainees are not deprived of rights or access to health care. No children should be newly detained and those in detention who can safely return to their families or communities should be prioritized for release with support for reintegration.^14^ The pandemic presents governments with an opportunity to reform justice delivery mechanisms, prioritize children’s cases and reduce the number of children deprived of their liberty.

### Norms and values

Pandemics can challenge or entrench harmful social norms relating to violence against children. Levels of violence against children may increase because of changes in the acceptability of emotional and physical violence at times of stress and insecurity. Disruption of informal networks of support from extended family members, friends and neighbours means that violence is less likely to be detected in private spaces during lockdown. Social norms can influence the prevalence of violence, help-seeking and prevention and response efforts. In India, greater increases in reports of intimate partner violence were found in districts with strict lockdown measures and where, before the pandemic, men reported wife-beating as justified.^15^ In Italy, a campaign to promote toll-free helplines for intimate partner violence was less effective in areas with strong norms condoning such violence.^16^ Strategies are needed to change social norms that support the acceptability of violence and sanction help-seeking to prevent and respond to violence against children. Examples include working with community leaders and using campaigns to increase the visibility of positive practices and attitudes.^17^ Social norms among decision-makers, governments and funding agencies may also affect the priority given to prevention of violence against children. Changing norms among these stakeholders could ensure violence against children is prioritized alongside other child health and protection issues.^18^

### Safe environments

The pandemic has changed the environments where children spend time, including public and community spaces, youth clubs and online.^19^ Online bullying, harassment and exploitation have increased.^20^ Increases in alcohol and firearms sales in many countries have contributed to unsafe environments, increasing the risk of abusive behaviours in households and threatening children’s safety.^21^^,^^22^ In some countries the risk of violence in public spaces, including police violence, has increased.^19^^,^^23^^,^^24^ Policies and efforts to promote safer community and digital environments for children include investing in community and youth organizations; limiting access to firearms and other weapons; and improved safeguarding for online spaces. Efforts are needed to prevent and address violence from police and government agencies, for example by preventing violent contact between young people and the police, reducing militarization of police and law enforcement agencies, and improving accountability for cases of violence against children. As local governments and cities rethink urban planning to limit and prevent the spread of COVID-19, there are opportunities to create child-responsive cities in consultation with young people, improve services for children and make built environments safe and inclusive for them.^25^

### Parent and caregiver support

Stress, anxiety and depression or threats to livelihoods during the pandemic can increase the risk of violence against children by caregivers and are also known risk factors for intimate partner violence.^26^^–^^28^ The COVID-19 pandemic has limited caregiver’s access to health workers, home-based parenting programmes, social services and social support. In response, efforts to support caregiver income, occupation and mental health should be prioritized. Paid childcare and sick leave, unemployment benefits, safe childcare support and access to health services, including mental health, should be made available to parents. Access needs to be ensured for families who lack digital technology.^29^^,^^30^ These strategies could be complemented by home- and community-based or online parent and caregiver programmes. Such programmes work with families to promote positive caregiver–child interactions, support nurturing care, engage fathers and strengthen referrals to health and protection services.^31^^,^^32^

### Income and economic strengthening

The impacts of the pandemic on poverty, household economic stress, and job and food security have been linked to increases in violence against children.^26^^,^^27^ As of May 2021, 222 countries and territories have planned or put in place 3333 social protection measures which include: income and economic support programmes (including cash transfers or universal basic income, housing support and social insurance) and active labour market policies.^33^ Although most programmes are targeted at households or adults, child-focused measures include: child grants, childcare subsidies and school meal programmes converted into take-home rations. Evidence from before the pandemic shows that cash transfers given primarily to women have strong protective effects on intimate partner violence and to a lesser extent on violence against children.^34^^,^^35^ A study in the United States of America found that domestic violence calls to the police during the pandemic decreased after government payments to individuals intended to stimulate the economy. The finding suggests that cash transfers can partially alleviate the financial stressors that trigger violence in the home.^36^ There is also evidence for the beneficial effects of social protection measures on food security, mental health and earnings during the pandemic.^37^ Efforts to improve child-sensitive social protection should include: universal targeting that prioritizes households with children; removing conditions on receipt of benefits and ensuring benefits are sufficient for household needs; and integrating complementary violence prevention programming or referrals into social protection programmes. 

### Response and support services

Disruptions to child protection services related to the pandemic have been documented in at least 104 countries.^38^ Reduced access to health, protective and legal services limits how caregivers and children can seek help, restricts the functioning of violence services for survivors, and affects the monitoring of child rights violations.^38^ Residential care facilities have also been affected, with evidence that children have been rapidly removed from care or unable to enter care.^39^ Services that are traditionally provided remotely, such as child helplines, have played a pivotal role in ensuring the continuation of child protection services.^40^ To ensure access, violence services should be designated as essential; funding and support for child helplines should be increased; and frontline staff should have safe working environments. Examples of adaptations of violence response services include: funding and supporting community-based organizations; training staff across different sectors to improve identification and referral for cases of violence against children; online case management; referral pathways which include access to relevant social welfare schemes; use of existing women’s spaces in refugee camps; and mobilization of youth networks and schools to share information about available services.^41^^,^^42^

### Education and life skills

The widespread closures of schools and universities during the pandemic has limited children’s access to teachers’ support, to peers and to school-based child protection resources.^43^^,^^44^ Given the important role of educators in identifying child abuse cases, a surge in child protection referrals may be expected as schools reopen. Concomitantly, teachers and learners may also be perpetrators of violence^3^ and are likely to be experiencing increased stress and anxiety, as well as potentially precarious conditions in school. These are all factors known to affect peer violence and the use of violent forms of discipline by teachers.^3^ Efforts to strengthen school-based child protection systems should include providing support and safeguarding training for teachers to identify and refer cases of violence, and offering psychosocial support to children.^13^^,^^44^ These efforts should be accompanied by long-term violence prevention and skills-building interventions aimed at creating gender-equitable, safe and healthy schools.^45^ Given that nearly 23.8 million additional children are estimated to be at risk of dropout from school,^44^ the effects of absenteeism on violence against children also need to be mitigated through back-to-school campaigns, subsidies and cash transfers given to mothers or caregivers.

## Looking ahead

Calls to strengthen child protection systems for violence against children long pre-date the current pandemic.^11^^,^^46^ The COVID-19 pandemic is a reminder of the essential role of governments, civil society and communities in improving funding, multisectoral coordination and data collection^47^ to address violence against children. Efforts need to be guided by the best interests of the child, by child developmental stages and by principles of equity and inclusion. To conclude, we offer five recommendations for policy-makers and practitioners to address violence against children in the context of the pandemic and beyond.

First, violence prevention and response services should be considered essential and unconditionally protected during, and beyond, pandemics. Budget allocation and human capital to address violence against children should be integrated into pandemic preparedness and disaster risk reduction strategies.^7^ The shift to remote delivery of services – child helplines, remote case management and the use of digital technologies – should be evaluated and expanded. Opportunities to integrate remote services into in-person service delivery should be explored.^40^ However, unless combined with efforts to reduce inequities in access to the internet and other technologies, relying solely on remote services will risk entrenching inequities in access to violence services. 

Second, the pandemic has further affirmed that violence prevention and response would not be possible without social workers, child protection staff, teachers and health workers. Policies to ensure labour rights, equitable benefits, gender equality and fair work environments for all categories of essential workers involved in violence prevention and response should be a priority for governments and international and local organizations.^42^

The pandemic response has underscored the importance of a public health approach to addressing violence with a focus on prevention at the structural, community, household and individual level.^8^^,^^9^ Our third recommendation therefore relates to the need for a multisectoral approach to violence prevention and response. Actions would require financial and political commitments from global stakeholders and governments to strengthen national and subnational child protection systems alongside improved global governance, financing and advocacy.^12^^,^^18^^,^^42^^,^^46^^,^^48^ A commitment across all sectors to the primary prevention of violence should aim towards addressing social inequality by investing in communities, schools, children and families. Both government and organizational policies are needed to support caregivers in employment, expand child-sensitive social protection programmes and embed violence prevention programmes in schools and in health and parenting interventions.^9^^,^^11^^,^^30^ Such an approach requires a commitment to financing, training and capacity-building from the education, health, and justice systems to improve help-seeking, service linkage and referrals, particularly for historically marginalized populations. Similarly, health workers, counsellors, teachers, community leaders and youth groups need to be prepared and resourced to identify and respond effectively to abuse and harm to children.^12^

Our fourth recommendation is that violence prevention should be guided by children and communities,^9^^,^^12^^,^^46^^,^^49^ as demonstrated by examples of young people leading social movements, and holding policy-makers to account. Opportunities to realize youth involvement should not be tokenistic or place the burden of changing systems, norms and practices solely on children. Instead, governments and institutions should enable children to use their agency and to lead and participate in the design and implementation of policy, programmes and research. Deeper partnerships and increased funding are needed for community organizations, indigenous leaders, women’s groups and faith-based organizations who played a role in reaching children during the pandemic – especially in the context of weak government responses.^46^

Finally, the pandemic has demonstrated the need for research and evidence to guide the primary prevention of violence and to mitigate both the short- and long-term sequalae of violence against children. Our final recommendation concerns future research – with children as leaders and partners^49^ – to focus on the structural determinants of violence against children to inform policy and action.^47^ The difficulty in estimating the prevalence and incidence of violence against children during the pandemic has underscored the need to improve efforts to collect real-time and disaggregated data to guide programmes and policies. Research is also needed on the effectiveness of large-scale interventions – including digital media, education interventions and response services – to prevent and respond to violence against children. This need for evidence is also mirrored in the gaps in the INSPIRE framework, which pays limited attention to the role of child agency, child participation and gender and social inequalities in experiences of violence and access to services. Any new evidence on violence against children will need to account for changes in how research is conducted, including the methodological and ethical challenges inherent in remote data collection. Research should always be accompanied by adequate safeguarding and referral pathways that involve local child protection providers, and prioritize the safety and well-being of children.^50^

The COVID-19 pandemic has changed how violence against children is experienced, reported and addressed, with long-term effects on children, adults and frontline workers. World leaders, United Nations agencies, activists, policy-makers and researchers have drawn attention to the harmful effects of the pandemic and called for multisectoral policies and programmes to respond to the acute and long-term needs of children. Prevention of violence against children should be included in COVID-19 response and recovery plans to ensure current and future generations of children do not suffer the long-term adverse health, social and economic outcomes associated with their experiences of violence.^8^^,^^11^
